# Socioeconomic inequalities in early childhood caries: evidence from vulnerable populations in Colombia

**DOI:** 10.1590/1807-3107bor-2024.vol38.0126

**Published:** 2024-12-09

**Authors:** Stefania Martignon, Carol C. Guarnizo-Herreño, Angela Maria Franco-Cortés, Lina Maria García-Zapata, Emilia Maria Ochoa-Acosta, Luis Fernando Restrepo-Pérez, Maria Cristina Arango, María del Pilar Cerezo, Andrea Cortes

**Affiliations:** (a) Universidad El Bosque, Caries Research Unit – Unica, Research Department, Bogotá, Colombia.; (b) Universidad Nacional de Colombia, Facultad de Odontología, Departamento de Salud Colectiva, Bogotá, Colombia.; (c) Universidad de Antioquia, Facultad de Odontología, Medellín, Colombia.; (d) Universidad del Valle, Facultad de Salud, Escuela de Odontología, Cali, Colombia.; (e) Universidad El Bosque, Facultad de Odontología, Bogotá, Colombia.; (f) Universidad Autonoma de Manizales, Programa de Odontología, Manizales, Colombia.

**Keywords:** Educational Status, Epidemiology, Social Class

## Abstract

The Colombian Chapter of the Alliance-for-a-Cavity-Free-Future (Col-ACFF) has been conducting a health promotion and caries prevention program among young children in four vulnerable Colombian municipalities (baseline data from 2012–2014). This study aimed to quantify socioeconomic inequalities in early childhood caries (ECC) and examine the potential role of daily fluoride-toothpaste use, previous-year dental-care visit, and nutrition/diet-related aspects. The study sample included 1344 children aged 1–5 years. Inequalities in the age-standardized prevalence rates of and mean number of tooth surfaces affected by moderate/extensive (d_ME_) and initial (d_IME_) caries (defined using the ICDAS-merged-epi criteria) by household income and level of education were examined using the relative index of inequality (RII) and the slope index of inequality (SII). Approximately one-third of the children included in this study exhibited d_ME_, while 84% exhibited d_IME_. The majority of outcomes exhibited social gradients, and significant relative (RII) and absolute (SII) inequalities in ECC were observed. The SII estimate indicated an absolute difference of 12.4% in the prevalence of moderate/extensive carious lesions among children living in households with the lowest compared to the highest education levels [SII: 12.4; 95% confidence interval (CI): 2.7–22.1]. These children were also 6.7 times more likely to exhibit d_IME_ compared to those living in households with higher levels of education (SII:6.73 95% CI: 4.18–9.29). Daily use of fluoride toothpastes, dental care visits in the previous year, and nutrition/diet-related factors played a limited role in ECC inequalities. In conclusion, significant ECC inequalities were observed in these vulnerable populations, highlighting the importance of upstream and downstream interventions that raise awareness among stakeholders and improve community- and individual-based practices to address this.

## Introduction

The caries experience in children has decreased globally over the past three decades. However, this reduction has not been universal, exhibiting a strong association with social and economic conditions and resulting in larger reductions in the caries burden of high-income countries.^1–5^ In addition to these country-level differences, evidence suggests that the incidence of caries in children is also associated with household socioeconomic characteristics^1^ such as income and parental education,^6^ which are part of a complex framework of contextual and individual factors^7^ that influence various aspects of oral health (e.g., access to oral healthcare products and preventive care; health literacy which affects oral health behaviors, service usage, and social participation).^6^ Children from disadvantaged backgrounds exhibit significantly higher prevalence of carious lesions and a greater mean number of affected tooth surfaces/teeth compared to those from higher socioeconomic backgrounds.^3,8^ Despite recent advancements, early childhood caries (ECC) remains a significant public health concern and academic and public policy challenge. In Colombia, the most recent National Oral Health Survey (ENSAB-IV-2014) showed a worrying increase in the prevalence of caries (dmft) from 60.4% in 1993 to 62.1% in 2014 [with the latter proportion increasing to 89% when initial (*i.e.*, non-cavitated) carious lesions (as per the ICDAS-merged criteria) were included] in children aged 5 years.^9^

ECC was recently defined in the Caries terminology consensus (2019) as "the early onset of caries in young children with often fast progression, which can finally result in complete destruction of the primary dentition."^10^ For epidemiological purposes, the agreed definition of ECC is "the presence of one or more decayed (non-cavitated or cavitated lesions), missing (due to caries), or filled surfaces in any primary tooth of a child under age of 6."^10^ ECC can affect the quality of life (QoL) of children and their families;^11,12^ negatively influence the child's growth and development;^13^ and significantly increase their risk of developing caries in the permanent dentition.^14^ Several studies have reported large variations in the incidence of ECC by household- and area-level socioeconomic position (SEP), ethnicity, and immigration status.^15–17^ Similar results have also been observed in countries with publicly funded universal oral healthcare services for children.^15,18^ Therefore, given its strong association with socioeconomic factors such as parental income and education levels,^19,20^ ECC may be considered as an early marker of social inequalities.^21^

Although the association between ECC and socioeconomic conditions has been studied extensively in various countries, there is limited evidence of the same in Colombia. Moreover, the majority of studies on inequalities in ECC have examined the whole socioeconomic spectrum of a society, and less is known about inequalities within certain vulnerable populations (e.g., unprotected individuals; communities facing difficulties due to limited resources, high levels of discrimination, and/or exclusion, thereby putting them at risk of harm and neglect^22^). A recent multilevel analysis of ENSAB-IV data in Colombia showed caries experience and untreated caries prevalence rates of 36.9% and 33.0%,^9^ respectively, in children aged 1, 3, and 5 years. Furthermore, a significant association between these two caries outcomes measures and age, low socioeconomic status, and use of the subsidized health insurance regimen was also observed.^23^ However, this study analyzed a nationally representative sample of children, while the current study aims to better understand the prevalence of inequalities within vulnerable populations specifically.

Like other developing countries, public oral health interventions for vulnerable populations in Colombia have primarily replicated traditional measures without taking the different oral health needs of these populations and the underlying social determinants of the same into consideration. To address this, four universities (i.e., Universidad El Bosque, Bogotá; Universidad de Antioquia, Medellín; Universidad del Valle, Cali; Universidad Auntónoma de Manizales, Manizales) joined forces with the Alliance for a Cavity-Free Future (ACFF) to develop the Colombian chapter of the ACFF (Col-ACFF). The members of Col-ACFF worked in conjunction with the universities, the local government representatives and communities of four vulnerable municipalities (i.e., Anapoima, Andes, Comuna 20-Cali, and Manizales) to establish agreements, with the aim of reducing the prevalence of ECC and improving oral health conditions in early childhood through the implementation of a locally adapted health promotion and caries prevention program.^9,24,25^ As per the ENSAB-IV-2014, the prevalence of caries in the primary teeth (dmf) of children aged 1, 3, and 5 years and living in these municipalities was 40%, 33%, 30%, and 33%, respectively.^9^

ECC is largely preventable and can significantly affect the child's general health, QoL, and future oral health, highlighting the importance of understanding the role of underlying factors such as SEP and how they operate under different contexts. This topic is largely under-researched in the Colombian population characterized by large socioeconomic inequalities; significant health and social consequences of long-lasting internal armed conflicts; and considerable barriers to accessing health and dental care. Moreover, understanding the potential role of different pathways linking SEP and ECC, particularly in vulnerable populations facing a higher burden of oral diseases, is essential to tackle inequalities effectively. Therefore, the current study aimed to quantify socioeconomic inequalities in ECC in vulnerable populations living in four Colombian municipalities, and examine the role of daily use of fluoride toothpaste, dental care visits in the previous year, and nutrition/diet-related factors in the same.

## Methodology

The study was approved by the ethical review committees of Universidad El Bosque (UEB2011149), Universidad de Antioquia (UdeA222013), Universidad Autónoma de Manizales (UAM40-2014), and Universidad del Valle (UV014-013).

### Data source and study sample

This observational study analyzed the baseline sociodemographic, oral health, and general health data (collected between 2012 and 2014 as part of the of Col-ACFF study) of all children aged 1–5-years and living in four Colombian municipalities (i.e., Anapoima, Andes, Comuna 20-Cali, Manizales).

Sample size calculation for the Col-ACFF study (including children aged 8 months to <6 years) was carried out using the formula for a single-group interventional study (Package "pwr") of children <6 years of age, taking the age–population distribution of each municipality (data obtained from the local government), a 5% significance level, and a 2% sampling error into consideration. The estimation yielded a final sample size of 1484 children [n = 316 for Anapoima^26^; n = 642 for Andes^27^; n = 102 for Comuna 20-Cali^28^; and n = 424 for Manizales^24^].

The local government of each municipality contacted the parents of eligible children via community health agents and the radio. The children of parents that signed the consent forms and gave permission for clinical examination, carried out on a first-come first-serve basis, were included in the study until the sample size was met.

The UNICA research group at Universidad El Bosque, who were previously qualified in the ICDAS Clinical Scoring System,^29,30^ trained and calibrated in a four-day theoretical and clinical course representative examiners from the three universities (inter-/intra-examiner reproducibility weighted kappa values ≥ 0.7), who then trained and calibrated 1–3 additional examiners in their dental schools.^29,30^ In each municipality, 1–4 examiners carried out caries assessment in the primary dentition (d) using the visual ICDAS-merged-epi criteria [i.e., d_I_: Initial, ICDAS 1–2 (non-cavitated caries); d_M_: Moderate, ICDAS 3–4; and d_E_: Extensive, ICDAS 5-6 (dentine/cavitated caries)] without air-drying. For these analyses, d_M_ and d_E_ were considered the WHO criteria for cavitated/dentinal carious lesions (d_ME_),^9^ while d_IME_ represented inclusion of initial lesions as well.

Trained research assistants shared interview-based questionnaires (modified from nationally validated tools) that collected information on 8 items including: a) household income; b) highest household education level; c) urban/rural residence; d) child's age; e) child's sex; f) daily use of fluoride toothpaste (confirming whether the toothpaste brand used contained the minimum fluoride concentration of ≥ 1000 ppm); g) dental care visits in the past year; and (8) breastfeeding history with the children's parents.^9,31^ Of these items, 1 and 2 were indicators of family SEP, while 8 was an indicator of diet and nutrition. The research assistants also recorded the child's height and weight to allow calculation of the height-for-age index (Table 1).^32^

**Table 1:** Baseline characteristics of the study sample (*i.e.*, children aged 1–5 years).

Characteristic			Territory				
			Anapoima (n = 263)	Andes (n = 586)	Comuna 20-Cali (n = 94)	Manizales (n = 401)	Total (n = 1,344)
Demographics							
	Age in years, mean (SD)		3.6 (1.3)	3.5 (1.4)	3.5 (1.0)	3.3 (1.1)	3.5 (1.3)
	Age in years, n (%)						
		≥1 to <2	36 (13.7)	99 (16.9)	16 (17.0)	105 (26.2)	256 (19.1)
		≥2 to <3	49 (18.6)	122 (20.8)	29 (30.8)	104 (25.9)	304 (22.6)
		≥3 to <4	75 (28.5)	115 (19.6)	28 (29.8)	121 (30.2)	339 (25.2)
		≥4 to <5	50 (19.0)	128 (21.9)	20 (21.3)	71 (17.7)	269 (20.0)
		≥5	53 (20.2)	122 (20.8)	1 (1.1)	0	176 (13.1)
	Place of residence, n (%)						
		Urban	140 (53.2)	181 (30.9)	85 (90.4)	401 (100)	807 (60.0)
		Rural	123 (46.8)	405 (69.1)	9 (9.6)	0	537 (40.0)
	Sex, n (%)						
		Female	155 (58.9)	275 (46.9)	39 (41.5)	183 (45.6)	652 (48.5)
		Male	108 (41.1)	311 (53.1)	55 (58.5)	218 (54.4)	692 (51.5)
	Education level, n (%)						
		None	7 (2.7)	219 (37.4)	7 (7.5)	29 (7.3)	262 (19.5)
		Primary	79 (30.0)	193 (32.9)	20 (21.3)	110 (27.4)	402 (29.9)
		Secondary	112 (42.6)	123 (21.0)	49 (52.1)	137 (34.2)	421 (31.3)
		Superior	65 (24.7)	51 (8.7)	18 (19.1)	125 (31.2)	259 (19.3)
	Income (MMW), n (%)						
		≤ ½	103 (39.2)	184 (31.4)	6 (6.4)	36 (9.0)	329 (24.5)
		> ½ to ≤ 1	129 (49.0)	295 (50.3)	41 (43.6)	190 (47.6)	655 (48.7)
		> 1 to ≤ 2	27 (10.3)	84 (14.3)	42 (44.7)	130 (32.4)	283 (21.1)
		> 2	4 (1.5)	23 (3.9)	5 (5.3)	45 (11.2)	77 (5.7)
Daily use of fluoride toothpaste							
	Daily use of fluoride-toothpaste, n (%)						
		No	54 (20.5)	72 (12.3)	1 (1.1)	267 (66.6)	394 (29.3)
		Yes	209 (79.55)	514 (87.7)	93 (98.9)	134 (33.4)	950 (70.7)
Nutrition/diet-related factors							
	Breastfeeding, n (%)						
		No	18 (6.8)	66 (11.3)	16 (17.0)	13 (3.2)	113 (8.4)
		Yes	245 (93.2)	520 (88.7)	78 (83.0)	388 (96.8)	1231 (91.6)
	Height-for-age						
		Adequate HA, n (%)	184 (70.0)	395 (67.4)	81 (86.2)	229 (57.1)	889 (66.2)
		Risk of low HA levels	58 (22.0)	147 (25.1)	10 (10.6)	47 (11.7)	262 (19.5)
		Low HA	21 (8.0)	44 (7.5)	3 (3.2)	125 (31.2)	193 (14.3)
Dental care visits in the previous year							
	Visited the dentist within the last year, n (%)						
		No	81 (30.8)	348 (59.4)	19 (20.2)	59 (14.7)	507 (37.7)
		Yes	182 (69.2)	238 (40.6)	75 (79.8)	342 (85.3)	837 (62.3)
Caries burden							
	Prevalence of dME, n (%)						
		0	189 (71.9)	374 (63.8)	69 (73.4)	276 (68.8)	908 (67.6)
		> 0	74 (28.1)	212 (36.2)	25 (26.6)	125 (31.2)	436 (32.4)
	Mean number of d_ME_ (SD)		1.76 (4.3)	2.81 (6.6)	2.05 (4.9)	2.22 (5.3)	2.37 (5.7)
	Prevalence of dIME, n (%)						
		0	100 (38.0)	55 (9.4)	24 (25.5)	35 (8.7)	214 (15.9)
		> 0	163 (62.0)	531 (90.6)	70 (74.5)	366 (91.3)	1130 (84.1)
		Mean number of d_IME_ (SD)	4.27 (6.51)	14.14 (14.31)	9.07 (9.82)	9.95 (11.10)	10.60 (12.43)

MMW: minimum monthly wage; SD: standard deviation; HA: height-for-age.

### Variables

Four outcome variables for two levels of caries severity were analyzed, including: a) d_ME_: presence/absence of and number of tooth surfaces (s) with Moderate-Extensive caries; and b) d_IME_: presence/absence of and number of tooth surfaces (s) with Initial-to-Extensive caries. Household income was recorded in four categories based on the Colombian monthly national minimum wage (NMW), as follows: (a) ≤ ½ NMW; (b) > half and ≤ 1 NMW; (c) > 1 and ≤ 2 NMW; and (d) > 2 NMW. Education was measured as the highest qualification attained by anyone in the household and was categorized into no formal education, primary, secondary, and superior (*i.e.*, technical or university) education. Covariates analyzed included the child's age, sex, and place of residence (*i.e*., urban/rural). Additionally, the potential roles of daily use of fluoride toothpaste, dental care visits in the previous year, breastfeeding history, and the height-for-age index in ECC inequalities was also examined. The first three factors were recorded as binary variables, while height-for-age index was categorized as normal, at risk, or low.

### Statistical analysis

The trends, prevalence, and mean values of each caries measure by the SEP indicators were estimated. The relative index of inequality (RII) and slope index of inequality (SII) were used to quantify inequalities in relative and absolute terms, respectively.^33,34^ Both measures consider all income and education groups (i.e., not just the highest and lowest groups) and are derived using robust Poisson regression and linear regression, respectively.^35^ The caries indicators were individually regressed on the SEP measures after adjusting for the above-mentioned covariates. Considering the requirements of the indices, the income and education categories were converted into quantitative scores using a 0–1 scale, with the values reflecting the mean proportion of the analytical sample having a higher SEP level. RII represented the ratio of caries prevalence in children in the lowest SEP to those in the highest SEP (values > 1 indicating higher caries levels among those with poorer socioeconomic characteristics), while SII represented to the absolute difference between the predicted caries outcomes of the highest and lowest income and education levels (values >0 demonstrating inequality). Finally, the daily use of fluoride toothpaste, dental care visits in the previous year, breastfeeding history, and the height-for-age index variables were included in the RII/SII models to allow exploration of their potential roles in the observed inequalities. The percentage reduction in the RII/SII coefficients were used to quantify the attenuation proportion for each adjustment using the following formula: $100\times (\beta_{0}-\beta_{1})/\beta_{0}$ [where β_0_ is the coefficient of the SEP variable in the initial base model and β_1_ is the coefficient of the SEP variable in a model including potential explanatory variable(s)]. The significance level was set at 5% and all analyses were performed using the statistical software, Stata® 12.0 (StataSE Corp LP, College Station, USA).

## Results

Of the 1484 children aged 8 months to <6 years included in the Col-ACFF study, 1344 children (Anapoima: n = 263; Andes: n = 586; Comuna 20-Cali: n = 94 and Manizales: n = 401) were included in the current study. Those aged < 1 year (n = 55) and those with incomplete questionnaire data and/or height/weight measures (n = 85, 5.9%) were excluded. Imputation was not considered necessary as the proportion of children with missing data was < 6%.^36,37^ The mean age of the study sample was 3.5 ± 1.3 years, and approximately 51.5% were boys (Table 1). The distribution of the study sample by place of residence (*i.e.*, rural/urban) varied across municipalities, with Comuna 20-Cali (n = 9) and Manizales (n = 0) reporting none/very few children from the rural areas. The household socioeconomic conditions also exhibited geographical differences, with lower educational levels being observed in Andes (*i.e.*, 70% with none/primary-level education in Andes vs. 29%–35% in the other three municipalities) and lower income levels (*i.e.*, ≤ 1 MMW) being observed in Andes (82%) and Anapoima (88%; compared to 50%–57% in the other two municipalities). Moderate/extensive carious lesions (d) were observed in one-third of the children, while initial-to-extensive carious lesions (d_IME_) were observed in 84%. The mean ± SD number of tooth surfaces affected were 2.4 ± 5.7 and 10.6 ± 12.4, respectively. The mean number of tooth surfaces affected by dental caries tended to be higher in the Andes and Manizales municipalities, while the prevalence of ECC was slightly higher in Comuna-20 Cali and Anapoima (Table 1).

A social gradient in the prevalence and mean values of the outcome measures by SEP was observed, with higher burdens being observed at lower education and income levels (Table 2). An exception to this pattern was observed for the prevalence of Initial-to-Extensive caries lesions, with estimates very similar across the three highest educational levels and followed an almost inverse gradient by income level.

**Table 2:** Prevalence rates and mean values of oral health outcome measures by socioeconomic position.

Variable	n	Prevalence of d_ME_		Prevalence of d_IME_		Mean number of d_ME_S (SD)		Mean number of d_IME_S (SD)	
	1344	n	%	n	%	n	%	n	%
None	262	100	38.2	240	91.6	2.9	7.2	14.3	14.2
Primary	402	141	35.1	333	82.8	2.7	5.8	11.5	12.9
Secondary	421	127	30.2	341	81.0	2.2	5.4	9.4	11.8
Superior	259	68	26.3	216	83.4	1.4	3.7	7.2	9.0
p-value		0.001		0.002		0.001		< 0.001	
< ½	329	117	35.6	271	82.4	3.1	7.7	12.1	14.6
> ½ to ≤ 1	655	224	34.2	556	84.9	2.3	5.1	11.0	12.2
> 1 a ≤ 2	283	82	29.0	236	83.4	1.8	4.7	8.8	10.4
> 2	77	13	16.9	67	87.0	0.7	2.8	6.4	8.0
p-value		0.002		0.474		0.001		0.001	

The RII and SII estimates indicated significant relative and absolute inequalities in ECC by education and income after adjusting for age, sex, and place of residence (Table 3). The SII estimate indicated an absolute difference of 12.4% in the prevalence of moderate/extensive carious lesions among children living in households with the lowest compared to the highest education levels [SII: 12.4; 95% confidence interval (CI): 2.7, 22.1]. Moreover, the number of tooth surfaces with initial-to-extensive carious lesions was 6.7 times higher in children living in households with the lowest compared to those with the highest education level. The RII estimates showed a two-fold increase in the number of carious surfaces when moving from the highest to the lowest levels of household education (RII: 1.89; 95%CI: 1.12–3.17 for moderate/extensive carious lesions; RII: 1.89; 95%CI: 1.48–2.42 for initial-to-extensive carious lesions), and a 2.4 times increase in the number of moderate/extensive carious surfaces when moving from the highest to the lowest income levels. The RII and SII values for the prevalence of initial-to-extensive carious lesions by income level were not statistically significant. Relative inequalities tended to be larger for the two Moderate-to-Extensive caries outcome measures compared to that of including the Initial caries outcome measures. The inequality gaps on absolute scales showed larger differences in the prevalence of moderate-to-extensive caries, whereas wider gaps were observed in the number of tooth surfaces affected by initial-to-extensive caries lesions.

**Table 3:** Relative and absolute inequalities in caries measures by education and income levels.

Variable		Prevalence of dME	Prevalence of d_IME_	Mean number of dMES	Mean number of d_IME_S
		(0: n = 908; > 0: n = 436)	(0: 214; > 0 : n = 1130)	2.37 ± 5.7	10.6 ± 12.4
RII (CI 95%)					
	Education level	1.46 (1.08–1.99)*	1.11 (1.01–1.21)*	1.89 (1.12–3.17)*	1.89 (1.48–2.42)***
	Income level	1.42 (1.06–1.90)*	0.95 (0.86–1.04)	2.42 (1.41–4.15)**	1.43 (1.12–1.82)**
SII (CI 95%)					
	Education level	12.4 (2.7–22.1)*	8.7 (1.2–16.3)*	1.49 (0.27–2.72)*	6.73 (4.18–9.29)***
	Income level	11.4 (1.9–20.8)*	4.2 (−12.0–3.5)	2.07 (0.74–3.40)*	3.71 (1.10–6.31)**

RII: Relative index of inequality; SII: Slope index of inequality; Models were adjusted for age, sex, and place of residence;

* p < 0.05;

** p < 0.01;

*** p < 0.001.

Negligible changes in the RII and SII estimates were observed upon including the daily use of fluoride toothpaste, dental care visits in the previous year, breastfeeding history, and the height-for-age index variables in the models, suggesting that these factors had a limited role in ECC inequalities by education (Table 4) and income (Table 5) levels. However, among these factors, daily use of fluoride toothpastes was seen to be associated with the largest reduction in inequality (up to 7.3%).

**Table 4:** Relative and absolute inequalities in caries measures by education level. Models were adjusted for potentially relevant factors.

A. Relative index of inequality (RII)	Prevalence of d_ME_	Attenuation %	Prevalence of d_IME_	Attenuation %	Mean number of d_ME_S	Attenuation %	Mean number of d_IME_S	Attenuation %
	RII (CI95%)		RII (CI95%)		RII (CI95%)		RII (CI95%)	
Base Model *	1.46 (1.08–1.99)		1.11 (1.01–1.21)		1.89 (1.12–3.17)		1.89 (1.48–2.42)	
Fluoridated toothpaste use	1.42 (1.05–1.94)	2.7	1.12 (1.02–1.23)	-0.9	1.84 (1.09–3.10)	2.6	1.85 (1.45–2.37)	2.1
Breastfeeding	1.46 (1.08–1.98)	0.0	1.10 (1.01–1.21)	0.9	1.89 (1.13–3.17)	0.0	1.89 (1.48–2.42)	0.0
Height-for-age	1.48 (1.09–2.02)	-1.4	1.11 (1.01–1.21)	0.0	1.93 (1.14–3.27)	-2.1	1.91 (1.49–2.45)	-1.1
Dental care visits in the previous year	1.47 (1.08–2.00)	-0.7	1.11 (1.01–1.21)	0.0	1.99 (1.17–3.40)	-5.3	1.90 (1.47–2.44)	-0.5
All	1.45 (1.06–1.99)	0.7	1.12 (1.02–1.23)	-0.9	2.00 (1.17–3.43)	-5.8	1.88 (1.46–2.42)	0.5
B. Slope index of inequality (SII)	Prevalence of d_ME_	Attenuation %	Prevalence of d_IME_	Attenuation %	Mean number of d_ME_S	Attenuation %	Mean number of d_IME_S	Attenuation %
	SII (CI95%)		SII (CI95%)		SII (CI95%)		SII (CI95%)	
Base Model *	12.4 (2.7–22.1)		8.7 (1.2–16.3)		1.49 (0.27–2.72)		6.73 (4.18–9.29)	
Fluoridated toothpaste use	11.5 (1.7–21.3)	7.3	9.7 (2.0–17.3)	-11.5	1.44 (0.21–2.67)	3.4	6.52 (3.96–9.07)	3.1
Breastfeeding	12.4 (2.7–22.0)	0.0	8.7 (1.1–16.2)	0.0	1.49 (0.27–2.72)	0.0	6.73 (4.17–9.28)	0.0
Height-for-age	12.7 (3.0–22.4)	-2.4	9.0 (1.4–16.6)	-3.4	1.53 (0.30–2.76)	-2.7	6.82 (4.27–9.37)	-1.3
Dental care visit in the previous year	12.5 (2.7–22.3)	-0.8	8.8 (1.2–16.4)	-1.1	1.62 (0.34–2.90)	-8.7	6.77 (4.17–9.38)	-0.6
All	12.1 (2.2–22.0)	2.4	9.8 (2.2–17.5)	-12.6	1.62 (0.34–2.90)	-8.7	6.68 (4.07–9.28)	0.7

Raw data for each variable shown in Table 1.

* Models were adjusted for age, sex, and residence zone.

**Table 5:** Relative and absolute inequalities in caries measures by income level. Models were adjusted for potentially relevant factors.

A. Relative index of inequality (RII)	Prevalence of d_ME_	Attenuation %	Prevalence of d_IME_	Attenuation %	Mean number of d_ME_S	Attenuation %	Mean number of d_IME_S	Attenuation %
	IRR (CI95%)		IRR (CI95%)		IRR (CI95%)		IRR (CI95%)	
Base Model *	1.42 (1.06–1.90)		0.95 (0.86–1.04)		2.42 (1.41–4.15)		1.43 (1.12–1.82)	
Fluoridated toothpaste use	1.39 (1.04–1.87)	2.1	0.95 (0.87–1.04)	0.0	2.38 (1.38–4.09)	1.6	1.40 (1.10–1.79)	2.1
Breastfeeding	1.43 (1.06–1.91)	-0.7	0.95 (0.87–1.04)	0.0	2.42 (1.40–4.17)	0.0	1.43 (1.12–1.83)	0.0
Height-for-age	1.40 (1.05–1.88)	1.4	0.94 (0.86–1.04)	1.0	2.38 (1.39–4.08)	1.6	1.42 (1.11–1.81)	0.7
Dental care visits in the previous year	1.42 (1.06–1.90)	0.0	0.95 (0.86–1.04)	0.0	2.49 (1.43–4.33)	-2.9	1.42 (1.11–1.82)	0.7
All	1.39 (1.03–1.86)	2.1	0.95 (0.86–1.04)	0.0	2.43 (1.40–4.22)	-0.4	1.40 (1.09–1.79)	2.1
B. Slope index of inequality (SII)	Prevalence of d_ME_	Attenuation %	Prevalence of d_IME_	Attenuation %	Mean number of d_ME_S	Attenuation %	Mean number of d_IME_S	Attenuation %
	SII (CI95%)		SII (CI95)		SII (CI95)		SII (CI95)	
Base Model *	11.4 (1.9–20.8)		–4.2 (-12.0–3.5)		2.07 (0.74–3.40)		3.71 (1.10–6.31)	
Fluoridated toothpaste use	10.8 (1.4–20.3)	5.3	-3.8 (-11.6–3.9)	9.5	2.04 (0.71–3.36)	1.4	3.55 (0.94–6.16)	4.3
Breastfeeding	11.5 (2.0–20.9)	-0.9	-3.9 (-11.6–3.7)	7.1	2.07 (0.73–3.40)	0.0	3.74 (1.12–6.35)	-0.8
Height-for-age	11.1 (1.7–20.5)	2.6	-4.4 (-12.2–3.3)	-4.8	2.04 (0.72–3.36)	1.4	3.64 (1.05–6.24)	1.9
Dental care visits in the previous year	11.4 (1.9–20.9)	0.0	-4.3 (-12.1–3.5)	-2.4%	2.14 (0.79–3.49)	-3.4%	3.69 (1.07–6.31)	0.5
All	10.8 (1.3–20.3)	5.3	-3.9 (-11.6–3.8)	7.1	2.08 (0.73–3.44)	-0.5	3.53 (0.90–6.15)	4.8

Raw data for each variable shown in Table 1.

* Models were adjusted for age, sex, and residence zone

## Discussion

The current study demonstrated ECC inequalities by household education and income levels in a relatively homogenously vulnerable population. These inequalities persisted even upon consideration of two cut-off points for defining caries using the ICDAS criteria. The magnitude of the inequalities tended to be larger for moderate-to-extensive compared to initial-to-extensive carious lesions. Daily use of fluoride toothpastes, dental care visits in the previous year, and factors related to nutrition and diet played a very limited role in the observed inequalities. Furthermore, a very high prevalence of ECC was observed, with 84% of the children exhibiting at least one primary tooth with initial-to-extensive carious lesions (d_IME_) and 32% exhibiting untreated moderate-to-extensive carious lesions (d_ME_).

No significant inequalities in the prevalence of Initial-to-Extensive carious lesions by income levels were observed in the current study, and this lack of a social gradient was not surprising given that most children in Colombia exhibit initial caries. A recent Colombian Oral Health Survey (2015) found that approximately 62% of children aged 1, 3, and 5 years presented with untreated initial-to-extensive carious lesions.^9^ The corresponding proportion in the current study was approximately 84%.

Significant socioeconomic inequalities in the number of tooth surfaces affected by carious lesions were observed, with values increasing by 3.7 and 6.7 tooth surfaces when moving from the highest to the lowest household income and education levels, respectively. This suggests that, although carious lesions are relatively common among Colombian children, the number of affected surfaces reflect the underlying socioeconomic conditions and, therefore, may be considered as an initial marker of vulnerability toward developing more extensive lesions.

Previous studies using national data^23^ as well as data from the Andes territory^27^ have demonstrated inequalities in the incidence of caries by social class, with approximately 75% of children from families with at least one member earning a fixed salary exhibiting caries compared to 91% of children from families with no fixed salary (p-value = 0.007). These findings highlight the importance of tailoring prevention strategies to reduce socioeconomic inequalities and addressing the structural determinants of oral health,^6–8^ with the relevance of the latter being recently corroborated (2021) by the 2031-FDI Vision which stated that oral diseases were both a cause and an effect of underlying poverty and social inequalities.^38^

In the current study, adjusting for dental care visits in the previous year, daily use of fluoride toothpastes, and nutrition and diet-related factors did not attenuate socioeconomic inequality. This could potentially be attributed to several factors including alternative pathways to inequalities (*e.g.*, materialist, psychosocial, and social relational) in this particular population, measurement issues, and data limitations. Breastfeeding, even if for a short period only, was recorded as a dichotomous variable,^39^ preventing examination of the influence of the duration of breastfeeding or age of introduction of sugary products on inequalities as suggested by a previous study.^40^ The lack of a rural population, which are expected to exhibit higher proportions of households with low income and education and, consequently, higher burden of caries (as per the most recent national oral health survey^9^), in two of the four municipalities included may also have affected the findings. An oral health birth cohort study would have greater potential in understanding ECC inequalities.^39^ Furthermore, data on the reason for and frequency of dental care visits in the previous year was also unavailable, preventing identification of any unfavorable patterns.^41^ The baseline Col-ACFF study also did not collect data on sugar consumption (which can negatively affect oral health);^41^ prevalence of other oral hygiene practices; or the frequency of using fluoride toothpastes (which have been shown to help control caries).^7,12,42^ Finally, although variations in behavioral, nutritional, and dietary factors by SEP could potentially explain ECC inequalities to a certain extent, some of these factors exhibited relatively weak associations with household education and income in the current study sample.

These findings were in agreement with previous studies that reported a higher risk of caries in young children from deprived backgrounds compared to those from more affluent backgrounds.^15,23,43–45^ A social gradient in ECC within vulnerable populations has also been reported previously, with a recent study observing a steep deprivation gradient in the caries experience of 4-year-old children from ethnic minority groups (e.g., Maori and Pacific origin) in New Zealand.^15^ In the Latin American context, some studies have confirmed that ECC is a sensitive marker of early-life socioeconomic disadvantage.^7,39,43,46^ This body of evidence, along with the findings of the current study, emphasize the urgent need for upstream interventions and socioeconomically tailored family-centric strategies within primary care settings aimed at decreasing inequalities in different contexts. The WHO recognizes that primary care teams (including community health agents) play a key role in the prevention and control of caries as their deep knowledge of the community allows them to provide permanent support to families and ensure continuity of health promotion and maintenance.^47^ ECC has also been shown to be associated with various life-course outcomes, suggesting that improving the oral health of young children can significantly contribute to improving their future health and well-being.^12^ As per the IAPD (International Association of Paediatric Dentistry) Bangkok Declaration, other preventive actions that can help reduce the prevalence of ECC indicators in this population include raising awareness among stakeholders groups; avoiding sugar intake before the age of 2 years; limiting sugar intake after the age of 2 years; toothbrushing twice daily using 1000 ppm fluoridated toothpaste; and visiting the dentist from the first year of life to ensure regular preventive dental care.^42^ It is relevant to highlight that previous interventions should trigger both the subject and the community, as many attend the dentist only with symptoms, so while preventive care would still be more beneficial at an individual base for regular dentist's attendants, it should not replace community-based or universal-coverage prevention,^8^ including providing access to essential medicines for children, such as silver diamine fluoride.^38,47^

The main strengths of this study include the use of different SEP indicators; two cut-off points to define caries using the ICDAS criteria; and the analysis of both absolute and relative inequalities. The limitations of this study include its cross-sectional design; inclusion of a study sample that is not fully representative of the underlying populations; and data limitations (*e.g.*, measurement issues, lack of data on potential explanatory factors etc.).

In conclusion, the current study showed that untreated caries in young children is a strong marker of social inequality, even within vulnerable populations. This poses significant challenges in terms of designing public policies and oral health improvement strategies. The findings also suggest that interventions promoting daily use of fluoride toothpastes, annual dental care visits, and improvement of nutrition/diet-related aspects may not be sufficient to tackle socioeconomic inequalities in ECC, and upstream interventions combined with socioeconomically tailored family-centric strategies within primary care settings should also be considered.
